# A DNA Vaccine Against Proadrenomedullin N-Terminal 20 Peptide (PAMP) Reduces Angiogenesis and Increases Lymphocyte and Macrophage Infiltration but Has No Effect on Tumor Burden in a Mouse Model of Lung Metastasis

**DOI:** 10.3390/vaccines13060586

**Published:** 2025-05-30

**Authors:** Tom Kalathil Raju, Srdan Tadic, Pablo Garrido, Laura Ochoa-Callejero, Judit Narro-Íñiguez, Josune García-Sanmartín, Alfredo Martínez

**Affiliations:** Angiogenesis Unit, Oncology Area, Center for Biomedical Research of La Rioja (CIBIR), 26006 Logroño, Spain; tkalathil@riojasalud.es (T.K.R.); srdant@riojasalud.es (S.T.); pgarridor@riojasalud.es (P.G.); locallejero@riojasalud.es (L.O.-C.); jnarro@riojasalud.es (J.N.-Í.); jgarcias@riojasalud.es (J.G.-S.)

**Keywords:** DNA vaccine, PAMP, angiogenesis, lymphocyte infiltration, macrophages, lung metastasis model, melanoma

## Abstract

**Background/Objectives:** Nucleic acid-based anticancer vaccines are becoming a very active field in the fight against cancer. Here, our goal was to generate an oral DNA vaccine targeting the angiogenic peptide, proadrenomedullin N-terminal 20 peptide (PAMP). **Methods:** An expression plasmid (PcPAMP) was generated by fusing the tetanus toxin epitopes P2 and P30 to the mouse PAMP sequence to counteract self-tolerance, and the empty plasmid was used as a negative control (PcNeg). The plasmids were introduced into *Salmonella typhimurium* bacteria that were then transformed into bacterial ghosts. C57BL/6J mice were orally immunized with the ghosts five times at 2-week intervals. Then, B16-F10 melanoma cells were injected into the tail vein to generate lung metastases. Furthermore, naïve CD4^+^ T cells were exposed to PAMP, and their secretome was analyzed by proximity extension assays. **Results:** Significant levels of anti-PAMP immunoglobulins were detected in the blood of PcPAMP-vaccinated mice and their levels of spleen CD8^+^ T cells were significantly higher than in those treated with PcNeg, indicating that self-tolerance was effectively broken. Although the number and size of lung metastases was similar between both experimental groups, there was a significant reduction in intratumoral angiogenesis and in cancer cell proliferation index in the PcPAMP group. Furthermore, these animals showed an intense infiltration of lymphocytes, including regulatory T cells, and M2-like macrophages into the metastases, that was not evident in the PcNeg group. In addition, PAMP induced upregulation of IL1β, IL6, IL7, IL12, IL27, TNFα, and FGF21, and downregulation of IL16 in naïve CD4^+^ T cells. **Conclusions:** Although the vaccine was not effective in reducing tumor growth, new proliferative and immune functions have been described for PAMP. These new functions include induction of melanoma proliferation and modulation of lymphocyte and macrophage tumor infiltration dynamics.

## 1. Introduction

Tumor angiogenesis constitutes one of the hallmarks of cancer and is an attractive target in cancer research since it is critical for tumor growth, invasion and metastasis [1]. Antiangiogenic therapies have been developed over the years and are a powerful clinical tool for the treatment of different malignancies [2,3]. Tumor angiogenesis is induced by the appearance of hypoxic areas that commonly form in solid tumors due to the continuous growth of the tumor, which pushes many cells away from the blood capillaries, thus reducing their access to nutrients and oxygen. The main cellular sensors for lack of oxygen are the hypoxia-inducible factors (HIFs). When oxygen tension drops below a certain threshold, HIF-1α expression increases in the cell cytoplasm and enters the nucleus to form a heterodimeric transcription factor with HIF-1β, and both together bind to specific DNA sequences (hypoxia response elements, or HREs) in the promotor region of specific survival genes, including those involved in angiogenesis, increasing their transactivation [4]. Although VEGF is the best-known angiogenic factor, there are many others, including angiopoietin 1 and 2, PDGF, bFGF, adrenomedullin (AM), and more [5,6]. All these factors activate specific receptors on the membrane of endothelial cells (ECs), leading to EC proliferation, migration, sprouting, adhesion, recruitment of additional cell types, and the formation of new vessels [7].

A little-known angiogenic factor is proadrenomedullin N-terminal 20 peptide (PAMP). This small peptide is coded by the proadrenomedullin gene (*Adm*), which is located in human chromosome 11 or mouse chromosome 7. The gene generates a 185 amino acid preprohormone that, after posttranslational modifications, generates two bioactive peptides, AM and PAMP, both of which need to be amidated at their carboxy end to exert their physiological activities [8]. As its name indicates, PAMP contains 20 amino acids that provide the peptide with a tight alpha helical shape [9]. Several functions have been associated with PAMP, including vasodilatation [10], bronchodilatation [11], inhibition of peripheral neural transmission [12], growth regulation [13], hormone secretion modulation [14], regulation of intestinal absorption [15], antimicrobial activity [16], and angiogenesis promotion [17]. Regarding angiogenesis, both in vitro and in vivo models show that PAMP is several orders of magnitude more potent than other angiogenic peptides, such as VEGF or AM, as it is able to induce angiogenesis at even femtomolar concentrations [17]. In addition, the peptide fragment PAMP_(12–20)_ acts as a specific inhibitor that can reduce angiogenesis and tumor growth [17].

Although the receptors for AM are well characterized [18], the exact nature of PAMP receptors is not clear. The Mas-related G protein-coupled receptor member X2 (MrgX2) [19] and the atypical chemokine receptor 3 (ACKR3) [20] have been proposed as potential receptors, although both of them show moderate affinity. Curiously, PAMP decorates the cytoskeleton of neurons and other cell types, contributing to regulating microtubule plasticity [21] and axonal transport [22], so some authors have suggested that the cytoskeleton may act as an intracellular PAMP “receptor” under some circumstances [23]. Anyhow, in the case of ECs, the addition of PAMP results in an inhibition of ATP-induced Ca^2+^ influx [17] in a similar manner to its physiological behavior in the adrenal gland [14].

Nucleic acid-based anticancer vaccines are becoming a very active field in the fight against cancer due to their safety, versatility, low generation price, and efficacy [24]. These vaccines may have a DNA or mRNA base, and some of them have already reached clinical trials, showing they are well tolerated, induce few adverse events, and result in high recurrence-free patient survival [25]. Furthermore, these vaccines are a great platform for generating personalized treatments since they can be rapidly tailored for specific neoantigens present in the patient’s tumor, even engaging artificial intelligence algorithms to optimize their design [26]. Nucleic acid-based vaccines have been investigated for several decades, and the experience of many laboratories has contributed to better understanding the needs for optimizing formulations, engineering, and the use of adjuvants and delivery strategies [27]. The idea of targeting angiogenesis with anticancer vaccines is not new, and many potential DNA vaccines have been tested in animal models [28], with a few of them having reached clinical trials [29,30,31,32]. Interestingly, all the antiangiogenic vaccines tested in clinical trials use the bacteria *Salmonella typhimurium* as an oral delivery carrier.

Therefore, given the impressive angiogenic potential of PAMP, we decided to build an oral DNA vaccine against this peptide and test its efficacy in a syngeneic mouse model of lung metastasis generated by melanoma cells, using *S. typhimurium* ghosts as carriers.

## 2. Materials and Methods

### 2.1. Cell Lines and Bacterial Strain

Mouse cell lines B16-F10 and Raw264.7 were acquired from the American Tissue Culture Collection (ATCC, Manassas, VA, USA) and were maintained in RPMI 1640 (Lonza, Basel, Switzerland) or DMEM (Gibco, Thermo Fisher Scientific, Waltham, MA, USA) media, respectively, supplemented with 10% fetal bovine serum (Gibco). The cultures were maintained under a humidified atmosphere of 95% air and 5% CO_2_ at 37 °C and sub-cultured before they became confluent using a 0.25% trypsin/EDTA solution (Lonza).

An attenuated bacterial strain Aro-/- and DAM-/- of *S. typhimurium* was generously provided by Prof. B.A.D. Stocker (Stanford University, Stanford, CA, USA) and maintained with LB broth (Sigma-Aldrich, St. Louis, MO, USA).

### 2.2. Plasmid Construction

**Figure 1:**
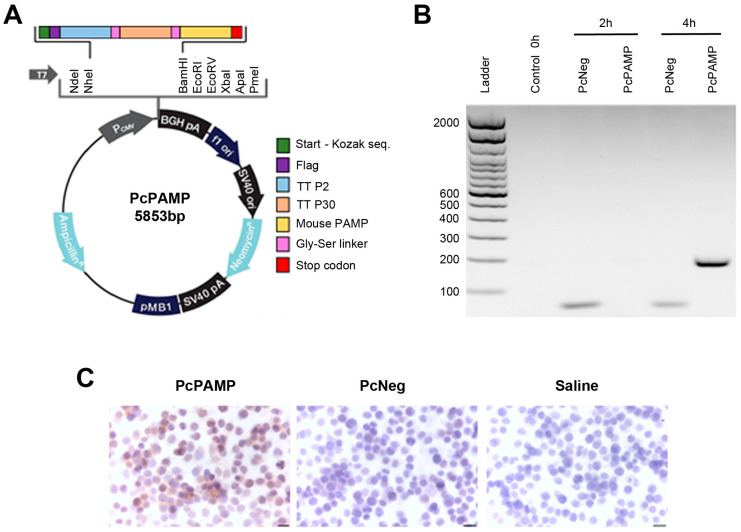
Construction and characterization of the vaccine. (**A**) PcPAMP plasmid vector map. (**B**) RT-PCR for the fusion peptide in Raw 264.7 macrophages before infection or after 2 h or 4 h of infection with ghosts containing either PcNeg or PcPAMP. (**C**) Immunocytochemistry with an antibody against mouse PAMP in Raw 264.7 cells infected with PcPAMP or PcNeg. The negative control was incubated with PBS. Scale bar = 20 µm.

A DNA fragment encoding the immune booster tetanus toxin (TT) epitopes P2 and P30 [33] bound to the amino end of murine PAMP through short linker regions [34] (see specific sequences in Table 1) was inserted in the PcDNA3.1 vector through custom synthesis (GenScript, Rijswijk, the Netherlands) and named PcPAMP (Figure 1A). The empty PcDNA3.1 plasmid was used as a negative control (PcNeg).

### 2.3. Preparation of Bacterial Ghosts

The plasmids were introduced into *S. typhimurium* through standard chemical transformation protocols, and transformed bacteria were selected with 0.1% ampicillin (Goldbio, St. Louis, MO, USA). Transformed bacteria were then converted into bacterial ghosts by treatment with 7% Tween-80 (Sigma-Aldrich) for 24 h, followed by lactic acid (Sigma-Aldrich) treatment for 60 min at pH 3.4, following published protocols [35].

### 2.4. Cell Transfection and Transcript Expression (RT-PCR and Immunocytochemistry)

Raw 264.7 cells (2.0 × 10^6^) were seeded and allowed to attach to the plate’s surface. Then, cells were exposed to 1.0 × 10^5^ bacterial ghosts containing either PcPAMP or PcNeg for periods of 2 or 4 h. Afterwards, the supernatant was removed and 500 µL Trizol (Invitrogen, Thermo Fisher Scientific, Waltham, MA, USA) were added to dissociate the cells. Cell extracts were stored at −80 °C until further use. For RNA extraction, 200 µL chloroform were added to the Trizol extracts and, after vigorous mixing and 3 min of incubation, the mixture was centrifuged at 12,000 rpm for 15 min at 4 °C. The middle white layer was transferred to a new tube and mixed with an equal volume of 96% ethanol (VWR chemicals, Radnor, PA, USA). The mixture was added to NZYSpin Binding Columns, and total RNA was isolated following the manufacturer’s instructions (MB13402, NZY total RNA isolation kit, NZYtech, Lisbon, Portugal). RNA was converted to cDNA by using a commercial kit (MB12501 NZY First-Strand cDNA synthesis kit, NZYtech) and the final 20 µL of cDNA was stored at −20 °C until further use. Using the cDNA as a template, standard PCR was performed using specific primers to detect the fusion peptide with sequences TT-PAMP_F: ACAAGGACGACGATGACAAG and TT-PAMP_R: GAACTGGCTGGGGGTGTC. The program consists of 94 °C for 3 min; 35 cycles of 94 °C for 30 s, 55 °C for 40 s and 72 °C for 60 s; and 72 °C for 7 min. PCR products were analyzed in 1.5% agarose gels in 1xTris-acetate-EDTA (TAE) buffer. A 100 bp DNA ladder (15628019, Invitrogen) was used for size reference.

### 2.5. Immunocytochemistry

Raw 264.7 cells (1.0 × 10^5^) were seeded on sterile glass coverslips in 6-well plates, incubated until they were 50–70% confluent, and exposed to *S. typhimurium* ghosts, as above. After thorough PBS rinses, the cells were fixed with 10% formalin for 10 min and permeabilized with 0.1% Triton X-100 in PBS for another 10 min. Following three washes with PBS, 10% goat serum in PBS was applied for 60 min to block non-specific binding. Then, cells were incubated with the primary anti-PAMP antibody at 4 °C overnight (Table 2). The following day, after three washes with PBS, cells were incubated with an HRP goat anti-rabbit secondary antibody (Table 2) for 60 min. After further washes with PBS, cells were developed with a diaminobenzidine (DAB) kit (K346811-2 Dako, Santa Clara, CA, USA) and lightly counterstained with Harris hematoxylin (Thermo Fisher Scientific), dehydrated, and coverslip mounted. Slides were observed and photographed with a DMI6000B microscope (Leica Biosystems, Nussloch, Germany).

### 2.6. Immunization Protocol and Lung Metastasis Model

All experiments and animal procedures were carried out following the guidelines laid down by the European Parliament and Counsel (2010/63/UE) and Spanish Royal Decree 53/2013 on animal experimentation, and were revised and approved by the CEAA/CIBIR Bioethics Committee (ref. AMR-17). The animals were maintained under specific pathogen-free (SPF) conditions inside ventilated racks at the CIBIR animal facility with a 12 h light/dark cycle and with food and water available ad libitum.

Seven-week-old C57BL/6J male mice (n = 20) were purchased from Charles River Laboratories (Sant Cugat del Vallès, Barcelona, Spain). The mice were randomly assigned to two experimental groups that were immunized with ghost cells containing either the PcPAMP or the PcNeg plasmid. Each animal received 2.0 × 10^6^ bacterial ghosts by oral gavage five times at two-week intervals. Two days after the fourth immunization, all mice were injected in the tail vein with 5.0 × 10^5^ B16-F10 melanoma cells, following our previously published protocol [36]. The mice were weighed periodically to detect any potential major adverse effect of the vaccine. Three weeks after tumor challenge, the mice were sacrificed, the number of lung metastases was assessed, and blood and tissues were collected.

### 2.7. Tissue Collection and Preparation

The mice were euthanized by intraperitoneal injection of 200 mg/Kg sodium pentobarbital (Dolethal, Vetoquinol, Madrid, Spain). Blood was collected by cardiac puncture, and serum was obtained by centrifugation at 1500× *g* for 10 min at 4 °C and stored at −80 °C until further use. The weight of individual lungs was recorded, and the number of metastases visible at the surface was assessed. The left pulmonary lobe was separated, snap-frozen in liquid nitrogen, and stored at −80 °C for future use. The right lung lobes were fixed by inflation with 4% paraformaldehyde for 24 h and then dehydrated and paraffin embedded (Excelsior AS Tissue Processor, Thermo Fisher Scientific) for histopathological analysis. Spleens were collected for splenocyte isolation and were analyzed by flow cytometry (see below).

### 2.8. Mouse ELISA

The humoral response to the PAMP vaccine was assessed by ELISA following our previous protocols [37]. Briefly, polystyrene 96-well plates (9017, Costar, Corning, New York, NY, USA) were incubated with 60 µL/well 5% glutaraldehyde (Sigma-Aldrich) in PBS for 60 min. After discarding the fixative, 60 µL/well containing 50 ng synthetic mouse PAMP (Proteogenix, Shiltigheim, France) was added and incubated overnight at 4 °C. The next day, the unbound peptide was removed and the plates were blocked with 150 µL/well of 1% bovine serum albumin (BSA, Sigma) in PBS that was incubated overnight at 4 °C. On the third day, BSA was discarded and 50 µL of mouse serum was added per well and incubated for 60 min at room temperature (RT). Mouse sera were analyzed in serial dilutions to establish the antibody titer for each animal. Positive controls were provided by a custom-made rabbit anti-mouse PAMP antibody (Proteogenix, Table 2) in serial dilutions, and negative controls contained just PBS. Following 3 vigorous washes with PBS, 50 µL/well of HRP-donkey anti-mouse IgG (Jackson Immunoresearch, West Grove, PA, USA) at a dilution of 1:10,000 was added for 60 min at RT. After a final set of washes, 50 µL/well of the substrate o-phenylenediamine dihydrochloride (OPD) (Sigma-Aldrich) was added and the reaction was stopped by addition of 3N HCl. Plates were scanned at 492 nm in a POLARstar microplate reader (BMG Labtech, Ortenberg, Germany).

### 2.9. Immunostaining Analyses

Immunohistochemical protocols were performed as described [38]. Tissue sections (3 μm thick) were dewaxed and rehydrated, and antigen retrieval was accomplished by heating with either 1.0 mM EDTA (pH 9.0) or 10 mM citrate (pH 6.0) for 20 min at 96 °C. After blocking with 10% normal donkey serum in PBS (Gibco) for 30 min, sections were incubated overnight at 4 °C with primary antibodies at the indicated concentration in 4% BSA in PBS (Table 2). The next day, following several washes in PBS, an HRP-secondary antibody or a polymer from either a Novolink (RE7200CE, Leica Biosystem, Nussloch, Germany) or an Immpress mouse system (MP-7402-50, Vector Labs, Newark, CA, USA) was added for 60 min at RT. Finally, the presence of HRP was developed with a DAB kit (Dako). The slides were lightly counterstained with Harris hematoxylin (Thermo Fisher Scientific), dehydrated, and permanently mounted. Photographs were taken with a Leica DMI6000B microscope (Leica Biosystems) that contains a digital camera. Random images (n = 10 per animal) were taken in the areas of interest, and Fiji imageJ v1.54p software was used to quantify the percentage of DAB-positive cells in each section. The value for each animal was the average of the ten random fields.

### 2.10. Splenocyte Isolation and Flow Cytometry Analysis

Mouse spleens were minced in small pieces and passed through a 70 µm cell strainer to separate the splenocytes. After discarding the supernatant, 1.0 mL of ACK lysis buffer (A10492, Gibco) was added to each spleen and incubated for 5 min at RT. Cells were washed twice with RPMI medium and then counted and resuspended in FACS buffer. Cell aliquots (1.0 × 10^6^ cells) were labeled with either anti-mouse CD4 antibody bound to APC/cyanine7 (100526, BioLegend, San Diego, CA, USA) or anti-mouse CD8 antibody bound to FITC (140404, BioLegend) for 30 min at 4 °C and analyzed in a flow cytometer (FACS Canto II, BD Bioscience, Franklin Lakes, NJ, USA).

### 2.11. PAMP Stimulation Assay and Secretome Analysis

Splenocytes were isolated from untreated mice as above, and their number was adjusted to 1.0 × 10^6^ cells/mL. Then, CD4^+^ lymphocytes were purified with the MojoSort CD4 T Cell Isolation Kit (480005, BioLegend) following the manufacturer’s instructions. The isolated T cells were kept in RPMI supplemented with 10% FBS, 1% penicillin–streptomycin, 50 μM 2-mercapto-ethanol, and 50 U/mL IL-2. Aliquots of 0.5 × 10^5^ CD4^+^ cells were incubated with synthetic mouse PAMP peptide at a concentration of 10 nM or 100 nM for 12 h at 37 °C. Then, the supernatants were collected and subjected to high-throughput real-time proteomics to simultaneously detect 48 mouse cytokines, using a proximity extension assay (Olink Target-48 mouse cytokine panel, Cobiomic Bioscience, Granada, Spain).

### 2.12. Statistical Analysis

Data sets that passed the normality test (Shapiro–Wilk) and showed variance homogeneity (Levene) were analyzed with parametric tests. Parametric tests included an unpaired Student’s *t*-test or two-way ANOVA using GraphPad Prism v8.0 for Windows (GraphPad Software, San Diego, CA, USA). Data that did not fulfill normality and/or variance homogeneity tests were analyzed by the two-tailed Wilcoxon rank test using Rstudio 2024.12.1 + 56.3.

Parametric data are expressed as mean ± SEM. Non-parametric data are presented as median ± distribution interval. A *p*-value < 0.05 was considered statistically significant.

## 3. Results

### 3.1. Plasmid Generation and Transcript Expression

Plasmid PcPAMP was generated by inserting the sequence of a fusion protein in the cloning site of commercial expression vector PcDNA3.1 (Figure 1A). The fusion protein contained the tetanus toxin epitopes P2 and P30 (as immune boosters) bound to the amino end of murine PAMP with short linkers in between (Figure 1A). In addition, the empty vector PcDNA3.1 was used as a negative control (PcNeg). The plasmids were inserted into *S. typhimurium* bacteria that were then transformed into bacterial ghosts to avoid infectivity. To ensure expression of the construct, mouse macrophage cell line Raw 264.7 was exposed to the ghosts and the mRNA expression of the fusion peptide was analyzed by RT-PCR. In the resulting gel, we saw a faint band of ~200 bp at 2 h that became much stronger in the PcPAMP-infected cells at 4 h after ghost exposure, indicating a neat overexpression of the construct’s mRNA (Figure 1B). To check for protein expression, an immunocytochemical analysis was performed with an in-house produced antibody against mouse PAMP. Some of the Raw 264.7 cells infected with PcPAMP ghosts showed a clear staining, while uninfected cells and cells infected with PcNeg ghosts had no staining (Figure 1C).

### 3.2. Immunization Campaign and Tumor Challenge

**Figure 2:**
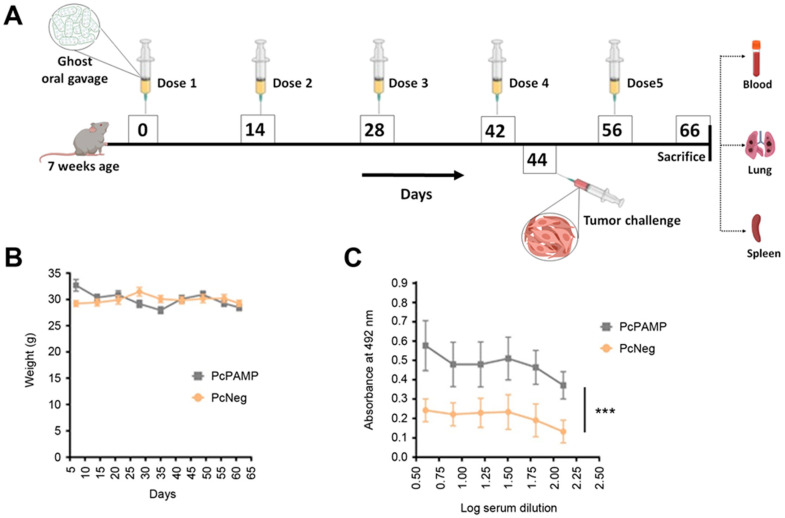
Experimental design, weight evolution, and antibody titers of experimental groups. (**A**) Schematic representation of the experimental protocol, showing periodical gavage of the oral vaccine and application of the tumor challenge. After sacrifice, blood and different tissues were collected. (**B**) Mouse weight was periodically taken during the treatment period. No significant differences were found. (**C**) ELISA quantification of anti-PAMP antibody titers on mice vaccinated with either PcPAMP or PcNeg ghosts. Two-way ANOVA. Each point represents the mean ± SEM of all animals in the group (n = 10 per group). ***: *p* < 0.001.

Mice were randomly divided into two experimental groups that received *S. typhimurium* ghosts containing either PcPAMP or PcNeg plasmids through oral gavage in five occasions at 2-week intervals (Figure 2A). Two days after the fourth immunization, a tumor challenge was performed by injecting B16-F10 melanoma cells into the tail vein as a lung metastasis model. Three weeks later, all animals were sacrificed, blood and tissues were collected, and the number of metastases was assessed (Figure 2A).

During the immunization period, the mouse weight was periodically checked as a surrogate of good health. No significant variations were observed, indicating a lack of major toxicity for the immunization treatment (Figure 2B).

Antibody titers were measured in mouse blood three weeks after the last immunization. Animals that received PcPAMP had significantly higher (two-way ANOVA, significant interaction F = 11.477, *p* < 0.001) titers of anti-PAMP antibodies than those treated with PcNeg (Figure 2C).

### 3.3. Lung Metastasis Quantification and Characterization of Cellular Immune Response

**Figure 3:**
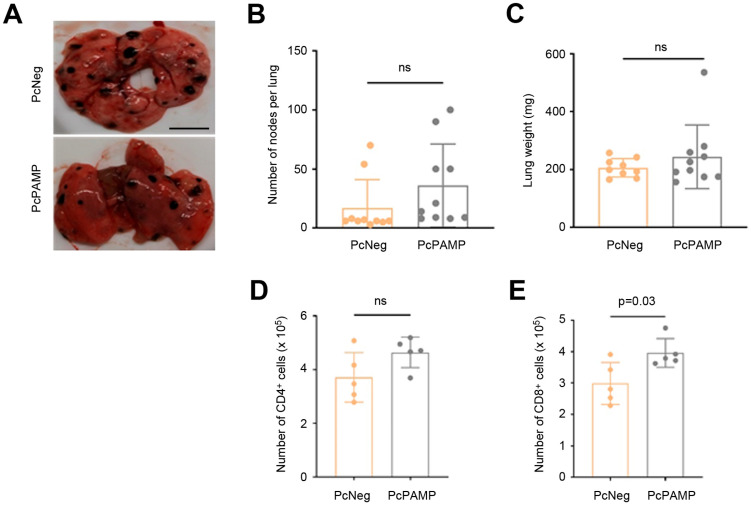
Analysis of metastases and splenocyte counts. (**A**) Representative photographs of mouse lungs belonging to both experimental groups. Melanoma metastases are easily seen as black nodules. Scale bar = 5 mm. Neither the number of metastases per lung (**B**) nor the weight of the lungs (**C**) was significantly different between groups. Splenocytes were labeled with CD4 (**D**) and CD8 (**E**) antibodies and analyzed by flow cytometry. Unpaired Student’s *t*-test. Each point represents the mean ± SEM of all animals in the group (n = 5–10 per group). ns = not significant.

Following mouse sacrifice, lungs were isolated and weighed, and the number of metastases was counted and recorded. All lungs had easily visible melanoma metastases (Figure 3A), but there were no statistically significant differences between the two experimental groups either in the number of metastases (Figure 3B) or in the total weight of the lungs (Figure 3C).

In order to investigate the influence of the vaccines on the cellular immune response, splenocytes were isolated from some animals, stained with antibodies against mouse CD4 and CD8, and quantified by flow cytometry (Supplementary Figure S1). There was no significant variation in the number of CD4^+^ lymphocytes (Figure 3D), but in the animals vaccinated with PcPAMP, there was a significantly higher (*p* = 0.03) number of CD8^+^ T cells than in those vaccinated with PcNeg (Figure 3E).

### 3.4. PAMP Vaccination Results in Less Angiogenesis and a Lower Number of Proliferating Tumor Cells in the Lung Metastases

Tissue sections from lungs belonging to both experimental groups were stained with antibodies against the endothelial cell marker CD31, the pericyte marker αSMA, and the proliferation marker Ki67. Marker expression was assessed both within the lung metastases and in the organ’s parenchyma.

**Figure 4:**
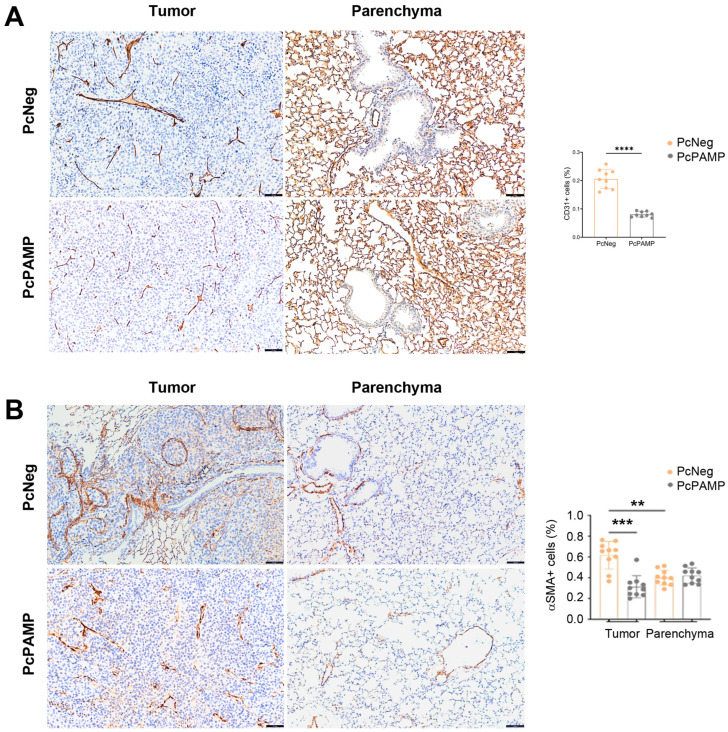
Angiogenesis analysis. Representative microphotographs of mouse lungs of both experimental groups stained with antibodies against CD31 (**A**) or αSMA (**B**). Images of the metastases (left column) and the parenchyma (right column) are shown. The percentage of positive cells was quantified by image analysis and is shown in the histograms. Since CD31 labels all endothelial cells in the alveoli, no quantification of the parenchyma was performed. Scale bars = 75 µm. Unpaired Student’s *t*-test. Each point represents the mean ± SEM of all animals in the group (n = 10 per group). **: *p* < 0.01; ***: *p* < 0.001; ****: *p* < 0.0001.

Regarding angiogenesis, as expected, we observed a much lower (*p* < 0.0001) number of CD31^+^ small blood vessels in the metastases of mice vaccinated with PcPAMP than in those receiving the control (PcNeg) (Figure 4A). Since CD31 labels all endothelial cells in the alveoli, it was impossible to quantify CD31 expression in the parenchyma (Figure 4A).

A similar pattern was observed for pericyte marker αSMA. First of all, there was an increased presence of αSMA^+^ pericytes within the metastases than in the parenchyma of the control animals (*p* = 0.001). Furthermore, in line with the main goal of the study, the number of pericytes (indicative of mature blood vessels) in the metastases of mice vaccinated with PcPAMP was significantly lower (*p* < 0.001) than in those vaccinated with PcNeg (Figure 4B). The number of mature blood vessels in the parenchyma was not affected, irrespective of the vaccination protocol.

**Figure 5:**
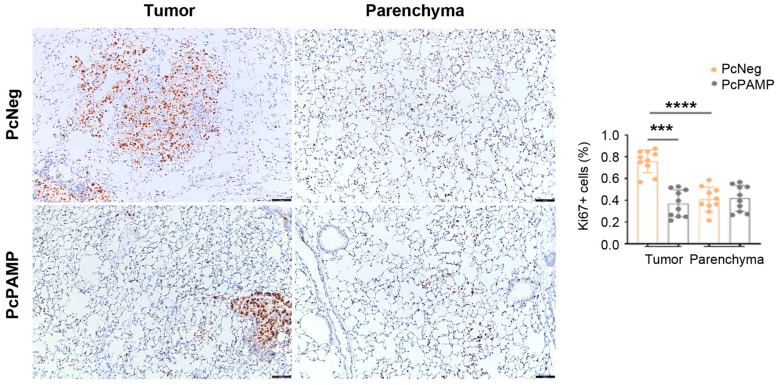
Analysis of tumor cell proliferation. Representative microphotographs of mouse lungs of both experimental groups stained with an antibody against Ki67. Images of the metastases (left column) and the parenchyma (right column) are shown. The percentage of positive cells was quantified by image analysis and is shown in the histogram. Scale bars = 75 µm. Unpaired Student’s *t*-test. Each point represents the mean ± SEM of all animals in the group (n = 10 per group). ***: *p* < 0.001; ****: *p* < 0.0001.

Similarly, the density of proliferating (Ki67^+^) cells was much higher (*p* < 0.0001) inside the tumor metastases than in the normal parenchyma of control mice. Interestingly, in the metastases of the animals that had been vaccinated with PcPAMP, the number of proliferating cells was significantly lower (*p* < 0.001) than in those vaccinated with PcNeg. Once again, the vaccination did not interfere with the proliferation of the normal tissue in the parenchyma (Figure 5).

### 3.5. PAMP Vaccination Results in a Massive Infiltration of Lymphocytes into the Lung Metastases

**Figure 6:**
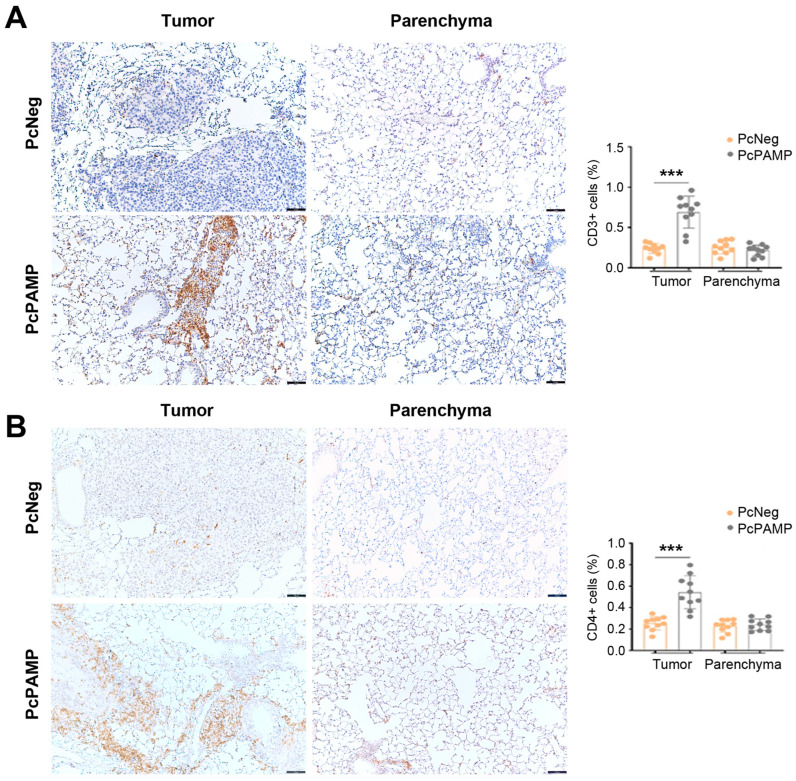
Tumor infiltration of CD3^+^ and CD4^+^ lymphocytes. Representative microphotographs of mouse lungs of both experimental groups stained with antibodies against CD3 (**A**) and CD4 (**B**). Images of the metastases (left column) and the parenchyma (right column) are shown. The percentage of positive cells was quantified by image analysis and is shown in the histograms. Scale bars = 75 µm. Unpaired Student’s *t*-test. Each point represents the mean ± SEM of all animals in the group (n = 10 per group). ***: *p* < 0.001.

**Figure 7:**
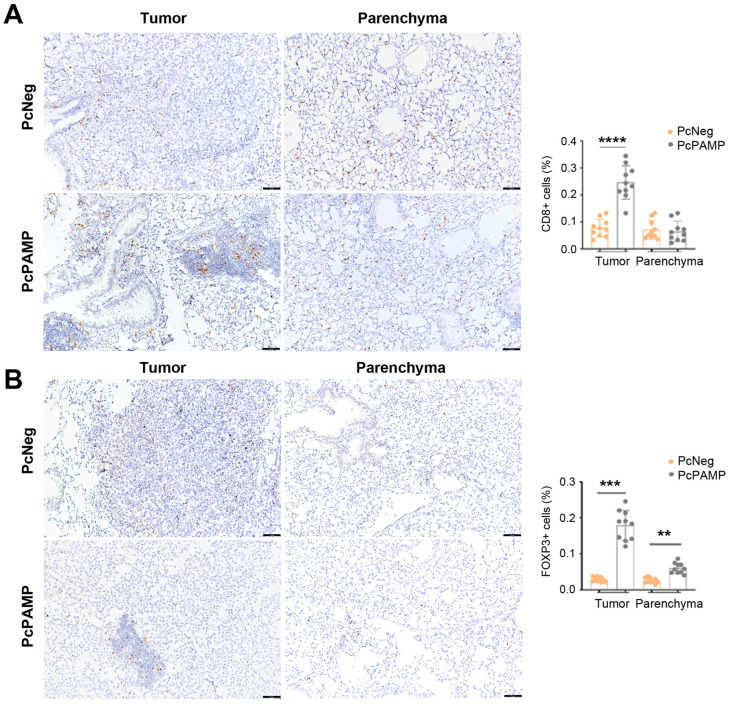
Tumor infiltration of CD8^+^ and FOXP3^+^ lymphocytes. Representative microphotographs of mouse lungs of both experimental groups stained with antibodies against CD8 (**A**) or FOXP3 (**B**). Images of the metastases (left column) and the parenchyma (right column) are shown. The percentage of positive cells was quantified by image analysis and is shown in the histograms. Scale bars = 75 µm. Unpaired Student’s *t*-test. Each point represents the mean ± SEM of all animals in the group (n = 10 per group). **: *p* < 0.01; ***: *p* < 0.001; ****: *p* < 0.0001.

Lymphocyte markers CD3, CD4, CD8, and FOXP3 were investigated in lung tissue sections of both experimental groups. For CD3, CD4, and CD8, the number of positive T cells in the metastases of the control animals (vaccinated with PcNeg) was undistinguishable from that found in the parenchyma. In contrast, the metastases of mice vaccinated with PcPAMP showed a much higher number (*p* < 0.001) of T lymphocytes compared with the metastases of the control group (Figure 6A,B and Figure 7A).

In the case of FOXP3, and similar to the other lymphocyte markers, there was a significant (*p* < 0.001) increase in positive cells inside the metastases of PcPAMP-vaccinated animals compared to controls. Interestingly, in contrast with the other lymphocyte markers, there was also a significant (*p* = 0.005) increase in the number of FOXP3^+^ cells in the parenchyma of immunized animals (Figure 7B).

### 3.6. PAMP Vaccination Results in a Higher Infiltration of Macrophages into the Lung Metastases

**Figure 8:**
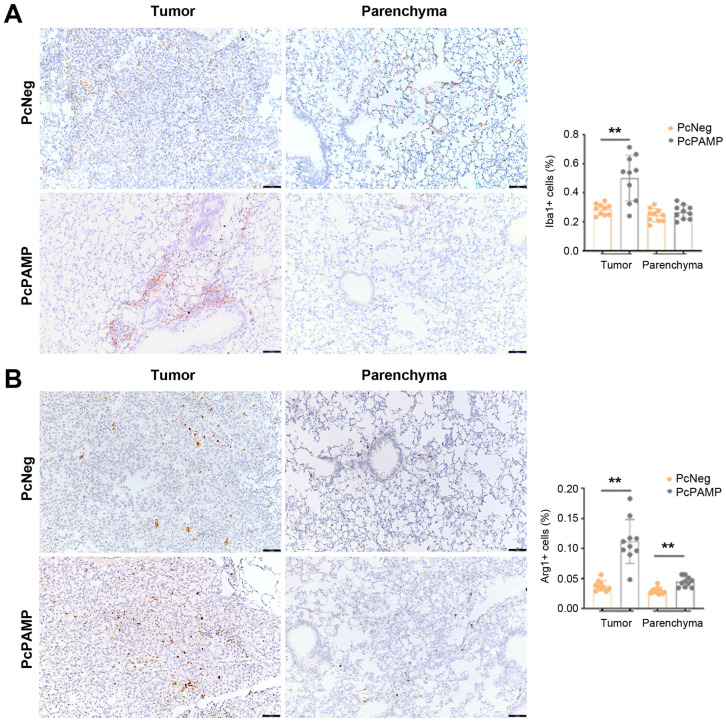
Tumor infiltration of macrophages. Representative microphotographs of mouse lungs of both experimental groups stained with antibodies against Iba1 (**A**) or Arg1 (**B**). Images of the metastases (left column) and the parenchyma (right column) are shown. The percentage of positive cells was quantified by image analysis and is shown in the histograms. Scale bars = 75 µm. Unpaired Student’s *t*-test. Each point represents the mean ± SEM of all animals in the group (n = 10 per group). **: *p* < 0.01.

Two macrophage markers were used in this study: ionized calcium-binding adapter molecule 1 (Iba1) and the enzyme arginase 1 (Arg1). Both markers showed an active recruitment of macrophages into the metastatic sites in the animals that received the PcPAMP vaccine compared with the control group (*p* = 0.002 in both cases). For Iba1 (Figure 8A), no changes were found in the parenchyma, but in the case of Arg1 (Figure 8B), a significant (*p* = 0.007) increase was found in the number of Arg1^+^ cells in the parenchyma of PcPAMP-vaccinated animals.

### 3.7. PAMP Modulates Cytokine Secretion in Mouse CD4^+^ T Cells

**Figure 9:**
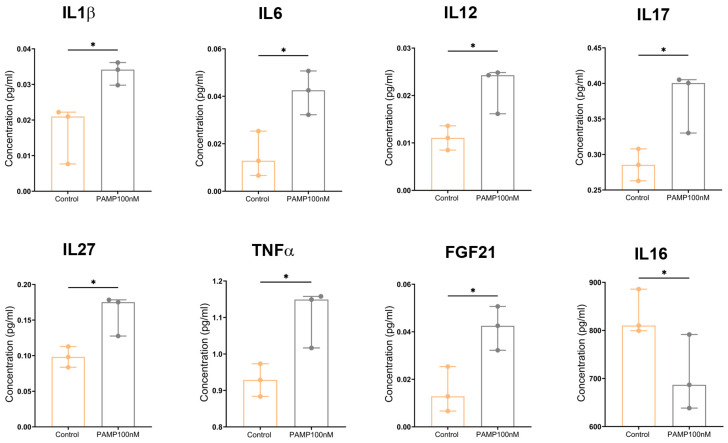
Proteomic analysis of the supernatants of CD4+ T cells exposed to 100 nM PAMP for 12 h. Two-tailed Wilcoxon rank test. Each point represents the median ± interval of all replicates (n = 3). *: *p* < 0.05.

To better understand the role of PAMP on lymphocyte dynamics and to demonstrate the presence of active PAMP receptors in these cells, naïve CD4^+^ mouse cells were exposed to either 10 nM or 100 nM synthetic PAMP for 12 h, and the supernatants were analyzed to investigate the lymphocyte’s secretome. Although some trends were found in the supernatants of lymphocytes exposed to 10 nM PAMP, none of those values were statistically significant. On the other hand, among the values of lymphocytes exposed to 100 nM PAMP, several significant differences were found. The soluble factors whose secretion was significantly (*p* < 0.05) increased after exposure to PAMP were IL1β, IL6, IL12, IL17, IL27, TNFα, and FGF21 (Figure 9). The only factor that showed a significantly reduced secretion after treatment was IL16 (Figure 9). Among the factors that did not show any significant difference were CXCL1, CXCL2, CXCL9, CXCL11, CCL2, CCL4, CCL5, CCl17, CCL22, IL4, IL5, IL7, IL9, IL21, IL22, CTLA4, CSF2, IFNα, IFNα2, IFNL2, PDCD1LG2, CD274, and HGF.

## 4. Discussion

In this study, an oral DNA vaccine was prepared targeting the angiogenic peptide PAMP. The vaccine was able to break tolerance to the self-antigen and produced specific antibodies against mouse PAMP, reduced tumor angiogenesis and tumor cell proliferation, and elevated levels of CD8^+^ T cells, but unfortunately, it was not able to reduce tumor burden. Nevertheless, important heretofore-unknown physiological functions of PAMP were revealed, including its participation in lymphocyte and macrophage homing into the tumor microenvironment.

When trying to generate an antiangiogenic vaccine against an endogenous factor, the problem of self-tolerance needs to be addressed. In our case, for our antigen, we used a chimeric protein that contained two tetanus toxin epitopes fused to murine PAMP. The tetanus toxin antigens have been previously employed in other vaccines as immune boosters [39,40], and since we obtained specific antibodies and an increase in the levels of CD8^+^ cells in the vaccinated animals, we understand that they were instrumental in overcoming the expected self-tolerance of the mouse immune system against the endogenously produced PAMP. In fact, the increase in CD8^+^ T cells we observed may have been an immune reaction to the tetanus antigens rather than to PAMP; however, the specific antibodies against PAMP found in vaccinated animals indicate the success of the vaccination strategy.

PAMP has been described as a potent angiogenic factor [17]; thus, we expected that a successful vaccine against PAMP should reduce tumor-related neovessel formation. This is exactly what we observed in the mice vaccinated with PcPAMP when we stained lung tissue sections with antibodies against CD31, an endothelial cell marker that is a good indicator of angiogenesis, or against αSMA, a pericyte marker that labels more mature vessels. Unfortunately, this reduction in the number of blood vessels did not translate into a decrease in the number of metastatic foci. The B16-F10 melanoma model is probably very aggressive [41], and the antiangiogenic therapy was not able to completely interfere with the mechanisms of metastasis formation. In fact, current clinical application of antiangiogenic drugs in cancer cases are never used as a single therapy but rather as part of a combination with chemo-, radio-, and/or immunotherapies [42]. Therefore, our antiPAMP vaccine will be tested in the future in combination with other drugs to fully understand its potential value. Furthermore, the large number of tumor-infiltrating lymphocytes and macrophages observed in the PcPAMP-vaccinated animals may have also played a role in generating a tolerogenic tumor microenvironment (see below).

We had a similar finding involving proliferation marker Ki67. Although AM has been widely characterized as an autocrine tumor cell growth factor in many malignancies [43,44], very few studies have focused on the potential effects of PAMP on tumor cell growth. An early report on neuroblastoma cell line TGW found that both AM and PAMP reduced the proliferation potential of this cell line [45]. Another study described the ability of AM to stimulate ^3^H-thymidine uptake on teratocarcinoma cell line PA1, whereas PAMP inhibited the AM-stimulated thymidine uptake in those cells [46]. Unfortunately, no studies are available with PAMP in other, more common cancer cell lines. Our current results indicate that vaccination against PAMP results in a significant decrease in the number of Ki67^+^ (proliferating) melanoma cells, pointing to a direct role of PAMP as a growth factor for this cancer of the skin. Future mechanistic studies are needed to elucidate whether PAMP acts as an autocrine, paracrine, or endocrine growth factor on these particular cells. Of course, another possibility is that the limited angiogenesis may be the cause of the reduction in tumor cell proliferation. As with the antiangiogenic effect, the lower proliferation index of the tumor cells did not translate into a significant reduction in the number of metastases.

An unexpected function of PAMP was its implication in lymphocyte and macrophage migration into the tumor microenvironment. The presence of immune cells within the tumor may have opposite effects. On the one hand, a high number of infiltrating lymphocytes is considered a survival marker for many tumors, including cutaneous melanoma [47,48], but on the other hand, many immune/inflammatory cells may induce both a chronic inflammatory state and immunosuppression. These cells include tumor-associated macrophages (TAMs), neutrophils, and myeloid-derived suppressor cells [49]. In our study, we found a high number of CD3^+^, CD4^+^, CD8^+^, and FOXP3^+^ cells within the metastases of the animals vaccinated with PcPAMP. The infiltration of CD3^+^ cells in cutaneous melanoma is usually described as a positive feature [50]. In the case of CD4^+^ cells, the landscape is a little bit more complex, since CD4^+^ T cells within the tumor can promote or suppress antitumor responses through the recognition of specific antigens. It seems that melanoma cells may exhaust cytotoxic CD4^+^ T cells through recognition of HLA class II-restricted neoantigens, and also through HLA class I-restricted tumor-associated antigens. This phenomenon may result in the stimulation of immunosuppressive CD4^+^ T_reg_ cells that may act as an immune evasion mechanism and favor tumor growth [51]. The presence of infiltrating CD8^+^ cells is also a complex matter. Apparently, CD8^+^ T cells initially infiltrate the tumor with an effector (antitumor) phenotype and are usually regarded as a positive feature for melanoma patients [52], but these cells rapidly transition into a dysfunctional state, which may contribute to the immunosuppressive environment of the tumor [53]. Finally, FOXP3 is a marker for T_reg_ cells, which are associated with negative outcomes for melanoma patients [52]. In any case, we recognize the limitations imposed by our immunohistochemical approach since the exact characterization of T_reg_ cells requires the simultaneous detection of many markers [54]. Anyhow, the large number of infiltrating lymphocytes elicited by the PcPAMP vaccine may be one of the reasons why, despite a reduction in angiogenesis and in the proliferation index of melanoma cells, we did not obtain a reduction in the number and/or size of metastases.

In a similar fashion to what we observed with lymphocytes, the metastases of PcPAMP-vaccinated mice also showed a large number of macrophages that were not present in PcNeg-vaccinated animals. In general, the presence of TAMs is regarded as a negative, protumoral feature that may induce angiogenesis and metastasis [55]. Two macrophage markers were used in our study: Iba1 and Arg1. Iba1 is expressed by all subpopulations of the monocyte/macrophage lineage [56] and labels both M1 and M2 macrophages. On the other hand, Arg1 is a marker of M2-like macrophages, which contribute to an immunosuppressive and tumorigenic environment [57]. The abundant presence of Arg1^+^ cells in the metastases of PcPAMP-vaccinated animals may provide further reasons to explain why we did not see a reduction in tumor metastases in our model.

Of course, some of these observations may have been due to the presence of the TT sequences in the vaccine’s antigen. Nevertheless, these immunoenhancing sequences have been used in many DNA vaccines, but none of these studies has described the massive infiltration of T cells and macrophages into the tumors we saw in our model [58,59], pointing to a direct involvement of PAMP.

There are several strategies to counteract a tolerogenic tumor microenvironment, including immune checkpoint blockade and adoptive cell therapies such as CAR-T, TCR Tg T cells, or ex vivo expanded TIL or NK cells [60]. Future experiments should investigate the combination of our vaccine with these novel therapeutic approaches.

Finally, to try to demonstrate a direct action of PAMP on mouse lymphocytes, naïve CD4^+^ T cells were exposed to synthetic PAMP for 12 h. Proteomic analysis of the supernatants demonstrated small but significant increases in several cytokines, while there was a significant decrease in IL16. Naïve CD4^+^ T cells can differentiate into T_H_1, T_H_2, T_H_9, T_H_17, T_H_22, and follicular effector T cells, as well as different subsets of T_reg_ cells, depending on their respective cytokine profile [61]. In this context, IL6 has been shown to direct leukocyte trafficking and T cell proliferation [62]; IL16 is a T cell-specific chemoattractant [63], and IL27 induces FOXP3 expression by T_reg_ cells [64]. All these functions could explain some of the phenotypic features we observed in the tumors following vaccination with the PAMP construct. Although our results suggest that lymphocytes may have functional PAMP receptors, additional molecular studies are needed to ascertain this interesting issue.

## 5. Conclusions

In summary, through our vaccination experiment, we have supported previously described functions of PAMP, such as its pro-angiogenic potential, and have discovered a number of new features, including its ability to induce melanoma cell growth and the regulation of lymphocyte and macrophage tumor-infiltration dynamics. While some of these functions may be mutually opposite in the regulation of tumor growth, this new knowledge opens new applications for the use of PAMP in tumor growth and immune cell modulation.

## Figures and Tables

**Table 1:** Sequence of gene fragments used to generate plasmid PcPAMP.

Gene Name	Sequence
TT P2 epitope	AAGCAAAAATATTTTAATGCAGTATATAAAAGCAAATTCTAAATTTATAGGTATAACTGAACTAAAAAAATTAGAATCAAA
TT P30 epitope	ATATTGAATATAATGATATGTTTAATAATTTTACCGTTAGCTTTTGGTTGAGGGTTCCTAAAGTATCTGCTAGTCATTTAGAACAATATGGCACAAATGAG
Mouse PAMP	GCAGGGCCAGATACTCCTTCGCAGTTCCGAAAGAAGTGGAATAAGTGGGCGCTAAGTCGTGCAGGGCCAGATACTCCTTCGCAGTTCCGAAAGAAGTGGAATAAGTGGGCGCTAAGTCGTGGGAAGCGA
Linkers	AGCGGCGGCGGCAGCGGCGGCGGCAGCGGCGGCGGCAGC

**Table 2:** Primary and secondary antibodies used for immunohistochemical analysis.

Primary Antibody	Reference	RRID	Dilution	Second Layer	Dilution of Second Layer
PAMP	16072 (Proteogenix, Schiltigheim, France)	AB_3694067	1:4000	7074 HRP goat anti-rabbit IgG (Cell Signaling, Danvers, MA, USA)	1:2000
CD31	ab281583 (Abcam, Cambridge, UK)	AB_3096925	1:4000	Novolink rabbit detection system	Prediluted
α-SMA	sc-53015 (Santa Cruz)	AB_628683	1:15,000	Immpress mouse detection system	Prediluted
Ki67	12202 (Cell Signaling)	AB_2620142	1:1000	Novolink rabbit detection system	Prediluted
CD3	A0452 (Dako, Santa Clara, CA, USA)	AB_2335677	1:1000	Novolink rabbit detection system	Prediluted
CD4	ab288724 (Abcam, Cambridge, UK)	AB_2941893	1:2000	Novolink rabbit detection system	Prediluted
CD8	ab217344 (Abcam)	AB_2890649	1:1000	Novolink rabbit detection system	Prediluted
FOXP3	14-5773-82 (Thermo Fisher)	AB_467576	1:100	112-035-003 HRP goat anti-rat (Jackson Immunoresearch, West Grove, PA, USA)	1:200
Iba1	sc-32725 (Santa Cruz, Dallas, TX, USA)	AB_667733	1:8000	Novolink mouse detection system	Prediluted
Arg1	93668 (Cell Signaling)	AB_2800207	1:1000	Novolink rabbit detection system	Prediluted

## Data Availability

All data are available in the main text or the Supplementary Materials.

## Supplementary Materials

The following supporting information can be downloaded at: https://www.mdpi.com/article/10.3390/vaccines13060586/s1, Figure S1: Representative flow cytometry results to study the cellular immune response.

## Abbreviations

The following abbreviations are used in this manuscript:

*Adm*	Proadrenomedullin gene
AM	Adrenomedullin
ACKR3	Atypical chemokine receptor 3
Arg1	Arginase 1
BSA	Bovine serum albumin
DAB	Diaminobenzidine
EC	Endothelial cells
HIF	Hypoxia-inducible factor
HRE	Hypoxia response element
HRP	Horseradish peroxidase
Iba1	Ionized calcium-binding adapter molecule 1
MrgX2	Mas-related G protein-coupled receptor member X2
PAMP	Proadrenomedullin N-terminal 20 peptide
SMA	Smooth muscle actin
TAM	Tumor-associated macrophage
TT	Tetanus toxin
