# Supplementary material for: A DNA Vaccine Against Proadrenomedullin N-Terminal 20 Peptide (PAMP) Reduces Angiogenesis and Increases Lymphocyte and Macrophage Infiltration but Has No Effect on Tumor Burden in a Mouse Model of Lung Metastasis
Source: Vaccines (Basel). 2025 May 30;13(6):586. doi: 10.3390/vaccines13060586 (PMC12197602; doi:10.3390/vaccines13060586)
Supplement: Supplementary file 1 [file vaccines-13-00586-s001.zip › vaccines-3634833-supplementary.pdf]

A DNA vaccine against Proadrenomedullin N-terminal 20 Peptide (PAMP) reduces angiogenesis and increases lymphocyte and macrophage infiltration but has no effect on tumor burden in a mouse model of lung metastasis

Tom Kalathil Raju [1], Srdan Tadic [1], Pablo Garrido [1], Laura Ochoa-Callejero [1], Judit Narro-Íñiguez [1], Josune García-Sanmartín [1] and Alfredo Martínez [1,*]

Supplementary material

Supplementary Figure S1

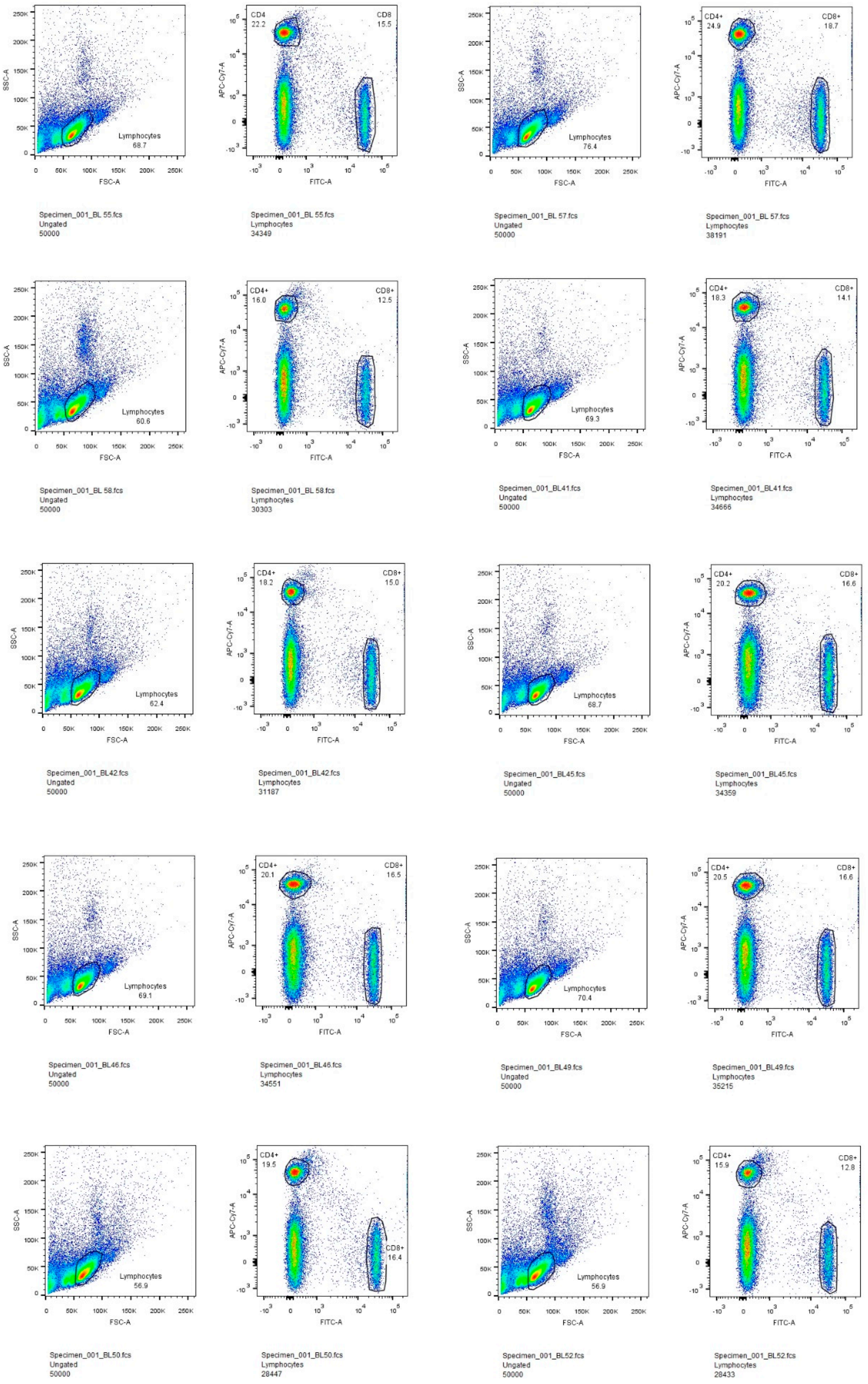

Supplementary Figure S1. Representative flow cytometry results to study the cellular immune response. Anti-mouse CD8 FITC and CD4 APC-Cy7 antibodies were used. The gating strategy was applied using FlowJo software and this layout shows representative results from control (n=5) and treated (n=5) animals. The gating strategy included gating on lymphocyte sub-population based on forward- (FSC) and side- (SSC) scatter and then separating CD8 FITC and CD4 APC-Cy7 positive cells.
